# Age and gender relations on LinkedIn pages of global staffing agencies

**DOI:** 10.1007/s10433-022-00726-x

**Published:** 2022-09-21

**Authors:** Wenqian Xu, Federica Previtali

**Affiliations:** 1 Division of Ageing and Social Change, Department of Culture and Society, Linköping University, 60174 Norrköping, Sweden; 2 Centre for Ageing and Supportive Environments, Lund University, Lund, Sweden; 3 Faculty of Social Sciences, Tampere University, Tampere, Finland; 4 Gerontology Researh Centre, Tampere, Finland

**Keywords:** Gendered ageism, Intersectionality, Older women, Older workers, Workforce diversity

## Abstract

This study investigates the ways in which age and gender play out on the LinkedIn pages of global staffing agencies through an intersectionality lens. A discourse analysis of 437 LinkedIn posts (including visual images, captions, and comments) was conducted. This study found that the corporate discourse of diversity shaped the ways that age and gender were represented. The portrayals of age and gender were found to create gendered specializations of labor and reproduce gender stereotypes; additionally, some workers were represented as disembodied clusters of attributes. The results of this study show the complex ways in which age and gender systems unfold, including two systems mutually reinforcing, gender/age system surfacing, and two systems dissolving. The findings suggest that diversity has lost its performativity as a concept, as its portrayals may not support disadvantaged groups gaining access to better employment opportunities. This study proposes that staffing agencies actively address intersectional disadvantages and foster a gender- and age-transformative change.

## Introduction

Age serves as a social organizing principle of nation states, personal biography and organizations (Hacking and Hacking 1990). McMullin (2000) argued that age dominates as the primary basis of difference and is a system of inequality. Drawing upon this sociological view of age stratification, older adults are often perceived and treated as being relatively disadvantaged, while being given lower status in terms of power, prestige, and influence (c.f. Mortimer and Moen 2016; Palmore 2016). Given that age shapes the ways that people value and treat different groups of people, Fineman (2014) called out the ageism found in some organizations that places value on youthfulness and prioritizes younger workers, thereby marginalizing older workers. Age as an organizing principle frames organizations and their practices in often implicit and hidden ways, despite legal protections against age discrimination in the workforce. The organizing power of age does not play out alone, but is embedded in an intricate net of power dynamics based on other categories, including gender, class, and ethnic relations.

Age and gender systems can interact to shape life situations and result in risks and disadvantages for women, leading to gendered ageism (Previtali et al. 2020). A UK study revealed that female staff in universities experienced discrimination on the grounds of age and gender, as well as their physical appearance (lookism), in a way that men did not experience (Granleese and Sayer 2006). A recent study also found that female staff in the private sector in Finland and Scotland experienced gendered ageism, as shown by comments concerning women’s roles, looks, sexual availability, possible pregnancy and menopause (Jyrkinen and McKie 2012). King et al. (2019) argued that an intersectionality perspective may be helpful for comprehending the intersections of multiple structures of inequalities, including age and gender. Given the interlocking nature of age and gender systems, Krekula et al. (2018) emphasized “the need to understand the powerful alongside the powerless” as a means of opening “a discourse of mutual shaping while recognizing the flexibility and the unfinished projects of creating differences” (p. 36). The interplay between the powerful and the powerless may manifest in ways that reinforce or weaken each other, possibly resulting in new forms of marginalization or offsetting (Krekula 2007). This study considers how age intersects with gender and how this intersection may create structures and inequality across professional life as a whole. In doing so, this paper joins the discussion on gendered ageism and intersecting inequality that people may experience in the workplace.

According to the media- and communication-centric perspective on social inequality, Trappel (2019) argued that media and communications have the power to both increase and reduce social inequalities in certain ways. With a focus on media content, McQuail (2010) argued that media gives more attention and prominence to men, treating business leaders more favorably than workers, while disregarding and stereotyping ethnic minorities and marginalizing the poor. Media content is often found to play a role in shaping and/or reflecting gender/age relations (e.gDjerf-Pierre and Edström 2020; Xu 2022). Looking at the ways that digital transformation affects society in increasingly profound ways, Curran (2002) argued that digital media may “undermine the hierarchical control of social knowledge by bypassing established mediating agencies and distributing restricted information” (p.55). Given the fact that digital media has been increasingly adopted by staffing agencies, our study focuses on age and gender systems in the digital media settings of staffing agencies.

Specifically, this study aims to explore how age and gender play out on the LinkedIn pages of global staffing agencies, using an intersectionality lens to shed light on the unique experiences of power relations and discrimination in the workplace. Two research questions (RQ) are addressed:

- RQ1: How are age and gender represented on the LinkedIn pages of global staffing agencies?
- RQ2: How do age and gender systems function in creating social inequality in the workplace?

To examine these questions, this study made use of a discourse analysis of 437 LinkedIn posts published over a two-year period (July 2019–June 2021) by five global staffing agencies, with headquarters in the Netherlands, the United Kingdom, and the USA. This study aims to contribute to the literature on gendered ageism in the workplace by exploring issues of power and status differences in visual media.

## Intersectionality and gendered ageism

The term “intersectionality” was coined by Kimberlé Crenshaw in 1989 to explain the oppression and discrimination of African American women. Intersectionality considers categories of age, gender, race, ethnicity, class, and other social positions to be interrelated and to mutually shape one another; it also explores the ways in which intersecting power relations influence social relations and individual experiences in everyday life (Collins and Bilge 2020). Intersectionality can be a useful analytical tool for understanding the varying experiences of individuals resulting from social divisions created by age, gender, and other factors. As noted by Collins (2015), “intersectionality is not simply a field of study to be mastered or an analytical strategy for understanding; rather, intersectionality as critical praxis sheds light on the doing of social justice work” (p. 16). Viewing issues through an intersectionality lens may hold the potential to advance fairness and equity in social institutions, as well as address discrimination on the basis of multiple discriminatory grounds.

This study adopts the perspective of intersectionality that views age and gender as interconnected and interdependent systems. Age and gender are often seen as two interactive power systems, which can lead to gendered ageism and increased inequality. Gendered ageism was introduced by Itzin and Phillipson (1993) in their study of age barriers in the workplace, where they focused on the experiences of women of all ages. The authors conceptualized age barriers as operating in the culture of organizations, while highlighting the intersecting effects of ageism and sexism on the organizational status and opportunities that women may have. Specifically, these barriers can manifest in women’s experiences of getting lower grades for their work, lacking access to timely training and having fewer career prospects (Itzin and Phillipson 2003). It should be noted that gendered ageism is empirically supported. For example, one study pointed out that widespread negative stereotypes, workplace policies and practices unfairly directed women to fulfill family care tasks (Barnett 2005). Another study investigating the effects of gendered ageism on women revealed that experiences of being unfairly forced out and facing unjustified obstacles when re-entering the workforce negatively impacted older women’s lives in terms of economic stability, self-esteem, social connectedness, and emotional well-being (Beaton 2018). Much of the research on gendered ageism remains theoretically underdeveloped and poorly explored, as gendered ageism appears to be reduced to discrimination against older women (Krekula 2007; Krekula et al. 2018; Previtali et al. 2020). In this way, it is important to extend the research to men and women of different age groups. As noted by Krekula (2007), the interplay between power systems manifests in the ways they reinforce or weaken one another, possibly resulting in new forms of marginalization or acting to offset each other. This study draws upon the theoretical notions of intersectionality to critically investigate social structures of age and gender in the workplace and the outcomes which these power structures jointly lead to. Moreover, intersectionality is used as an analytical tool to examine the different ways that heterogeneous members of specific categories (women/men) are represented in the workplace depending on their age, in addition to looking at inter-categorical differences.

## Diversity and inclusion (D&I) initiatives in global staffing agencies

Global staffing agencies are external intermediaries that help organizations fulfill human resource (HR) functions, including recruiting employees and improving HR managerial practices. Given that an increasing number of companies have outsourced recruiting functions and other HR-related matters, staffing agencies have gained increasing importance as actors in the labor market and in establishing professional relationships. They must operate under the constant tension of pleasing two types of customers: outsourcing companies and the talents that they wish to attract (Wears and Fisher 2012). Given that age discrimination often occurs in the hiring process (Cebola et al. 2021), staffing agencies can play a positive role in terms of tackling ageism and fostering a diverse and inclusive workplace.

Fostering a diverse and inclusive workplace has become a topical trend in the field of HR consultancy and practice. Diversity in regards to age, cultural background, disability status, gender, race and ethnicity, and sexual orientation has been established as a managerial issue in the workplace. A recent survey by Staffing Industry Analysts (2020) showed that almost two-thirds of workforce leaders across the globe see Diversity and Inclusion (D&I) efforts for their employed workforce as a priority. Staffing agencies tend to necessitate employing a diversified workforce and accentuate the strength of workforce diversity in terms of improving the productivity of an organization if managed appropriately. There are numerous examples of efforts made by global staffing agencies to support workforce diversity, including through D&I initiatives. However, the effects of D&I campaigns in terms of promoting inclusion and diversity in organizations need to be approached with a critical mindset. For example, one Israeli study found that D&I initiatives taken by staffing agencies can lead to the “othering” of minority job candidates and may increase work precarity (Kuna and Nadiv 2019).

As noted by Fuchs (2013), global companies have three dimensions of business power, namely business instrumental, structural, and discursive power, with different sources of power and channels through which power is exercised. Global companies are seen as being constituted by discursive struggles and competing efforts of sense-making, with the ability to enhance or challenge established power relations (Geppert et al. 2016). Furthermore, their digital media activity pages embody the power inherent in the selection of texts, discursive features, and content posting (as sociocultural practices and contexts). Global companies can be seen as one sort of institution whose practices lead to special meanings for the things placed within it.

The discursive practices of singling out certain persons, labeling them, and hence othering them, have been identified as reinforcing stereotypes about targeted minorities and downgrading them. This managerial discourse is found to have an ageist effect on older workers (Previtali et al. 2020). Similarly, a UK study found that the diversity framework tends to reinforce some single-strand identity categories, which can homogenize groups and overlook the complex interplay of individual lives and needs. The authors of this study explored the ways that diversity was practiced by third-sector organizations that tackled women’s diversified needs, showing that diversity “affects public feelings, making organizations and institutions feel better for using the signifier of difference in certain ways” (Vacchelli and Mesari, 2021; p. 431). Age diversity as part of the policy and institutional and managerial discourse of diversity is a recent addition, coming amid greater awareness of an aging workforce and longer working lives in countries with increased longevity. As suggested by critical studies (Krekula and Vickerstaff 2020; Previtali et al. 2020), policies on creating an age-diverse workforce run the risk of creating one ideal older worker by homogenizing older adults and forcing the attributes of life-stage groups on all the others. As for the concept of diversity in the context of this study, this paper examines the role that age and gender play in digital media produced by global staffing agencies by analyzing their respective practices when it comes to representing the meanings of age and gender.

## Data and method

Our study focuses on the pages managed by well-established global staffing agencies using LinkedIn, a digital platform offering employment-oriented online services where workers can update their CVs and career news, and corporate users can advertise jobs and corporate events. LinkedIn, in its capacity as a social media site, enables online interactions between corporate users (e.g., staffing agencies) and workers. For this study, a number of influential and dominant global staffing agencies that use LinkedIn corporate accounts were selected (Table 1). These agencies are headquartered in the Netherlands, the United Kingdom and the USA; their names were anonymized for ethical reasons. To the best knowledge of the authors, this study setting has not been seen in previous research and therefore may be valuable for contributing to understanding the role of age and gender in the workplace.

**Table 1:** List of selected global staffing agencies

Staffing agencies	Company scale (Number of employees)	LinkedIn followers (*n*)	Market share (%)
R	57,805	1,300,000 +	5
M	63,722	1,900,000 +	4
H	22,008	4,800,000 +	2
B	32,572	1,300,000 +	1
K	10,771	1,100,000 +	1

This study established a data corpus of 437 LinkedIn posts with visual images that portray age and gender in the workplace (Table 2). Specifically, all available posts portraying people of different ages and genders were collected. To ensure a timely appraisal, collected posts were limited to a 2-year period (July 2019–June 2021). Most of the posts included a single visual image, descriptive caption, and a few comments. The data corpus was safely stored in the online spaces of authors’ university institutions.

**Table 2:** Data corpus of LinkedIn posts

Staffing agencies	LinkedIn posts (*n*)	Posts including photos of women	Posts including photographs of men
R	83	44	59
M	61	48	37
H	128	99	64
B	16	10	10
K	149	115	64
Total	437	316	234

As the primary data material for this study, it should be noted that visual images can provide abundant information about age and gender. From a visual communication perspective, visual images (re-)produce meanings in our society; images of certain social groups may be vital in the context of social justice and balance of power (Loos and Ivan 2018; Xu 2019; Ylänne 2015). Ageist stereotypes in the media (including visual media) can negatively affect older people’s self-esteem, as well as their cognitive and physical health (Levy et al. 2002a, b; Levy et al. 2002a, b). This study acknowledged the need to carefully analyze visual images in the data corpus alongside the analysis of supplementary captions and comments.

**Fig. 1:**
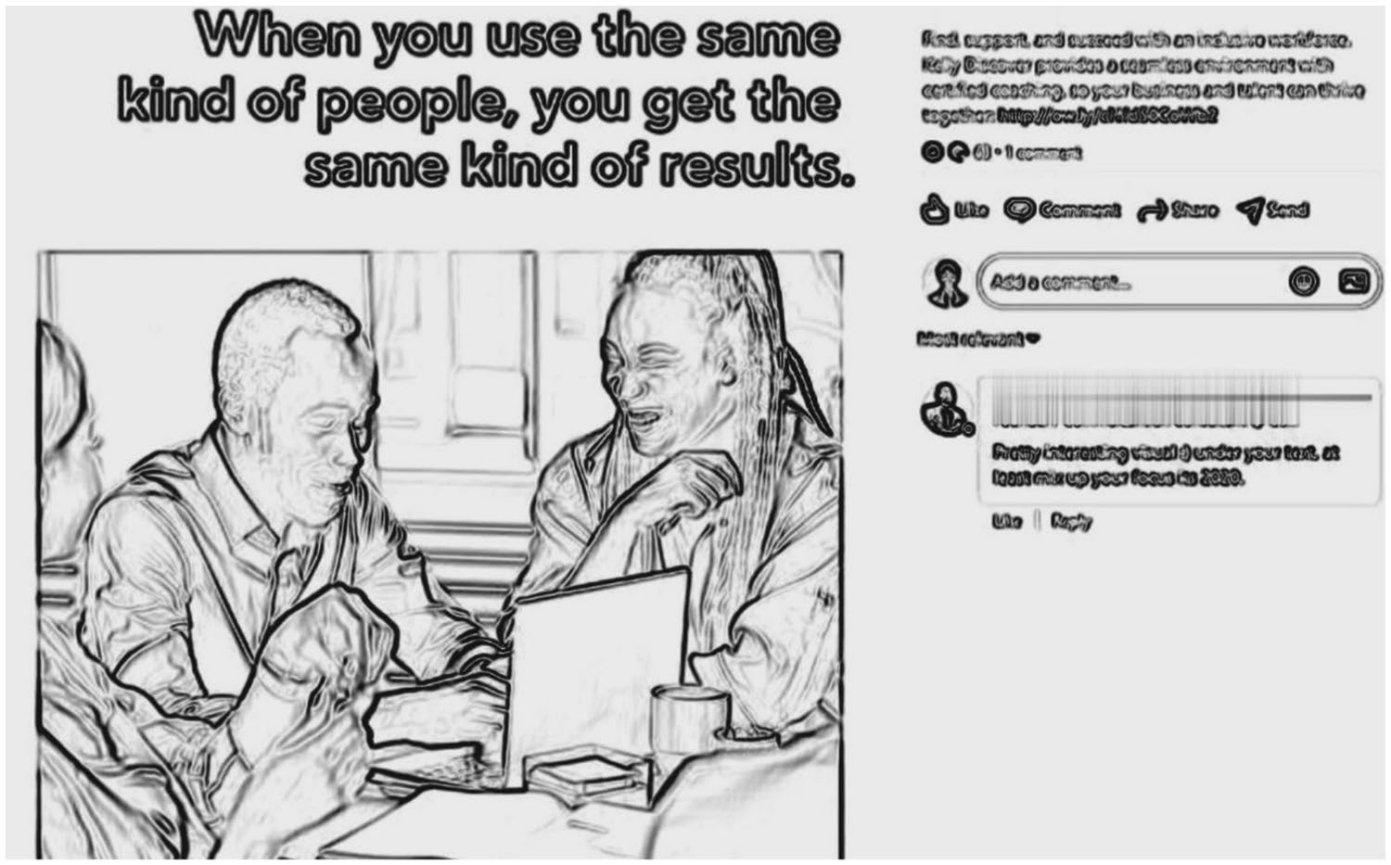
Sample unit of analysis. *Headline: When you use the same kind of people, you get the same kind of results. Caption: Find support and succeed with an inclusive workforce. [Agency] provides a seamless environment with certified coaching, so your business and talent can thrive together. [webpage link]. (63 likes and celebration, 1 comment). Comment: Pretty interesting visual:) under your text, at least mix up your focus in 2020. (no likes, no replies)*

In this study, discourse analysis of LinkedIn posts published by the five staffing agencies was conducted. Figure 1 displays an analytical unit of this study, namely a LinkedIn post including a visual image, post caption, and comments. As a method of visual image analysis, discourse analysis is useful for examining images and interpreting their effects, especially concerning constructions of social difference (Rose 2016). This study draws upon the method of discourse analysis with visual materials developed by Gillian Rose (2016), given that the method pays more attention to the material practices of institutions than it does to visual images. This method is based on Foucauldian notions of discourse, indicating that “the dominant discourse occurs not only because they were located in socially powerful institutions but also because their discourses claimed absolute truth” (Rose 2016, p. 190). Based on Foucault’s views on the ways that institutions work through apparatuses and technologies, Rose (2016) defined institutional apparatuses as the forms of power/knowledge that constitute institutions, while seeing institutional technologies as practical techniques used to put that power/knowledge into practice. This type of analysis focusing on institutional apparatuses and technologies concerns the production and use of visual images, rather than the details of single images (Rose 2016). It is useful for generating empirical accounts for given texts and institutions, as well as established routines and practices, despite having limitations in terms of reflexivity (for a methodological critique, see Rose 2016). In this study, visual images are seen as articulations of institutional power.

This study analyzed visual images, captions, and comments (if any), and how they interact with one another. As the first step in the analysis, visual images, written texts, posting practices and the interactions between these elements were viewed and re-viewed in order to obtain an understanding of the materials. In the second phase, an assemblage of analytical concepts was established for the purpose of coding the data, based on Rose’s (2016) concepts of institutional apparatuses and institutional technologies. For the analytical process, focus was placed on the content, production, and use of LinkedIn posts. The critical questions include.

- *Content*: In what ways does LinkedIn portray of age and gender?
- *Production*: What forms of knowledge can be seen (e.g., scientific literature, corporate policies, industry guidelines), and what discourses are articulated?
- *Use*: What practical techniques are used to construct age/gender relations? (with a focus on visual design and posting practices)
- *Audiencing*: Did LinkedIn users critique the knowledge/power articulated by staffing agencies, and did LinkedIn activities allow users to engage effectively?

This analysis was performed by having the first author complete the coding and the second author carefully follow up on the whole process. In the third step, codes were analyzed to identify the dominant ways in which heterogeneous women/men are represented differently depending on their age, and what inter-categorical sex differences can be seen among young/older people. In this process, divergent opinions concerning codes and categorizations were discussed, the adequacy of the analysis was checked and relevant complements were added. Table 3 presents an example of the analytical process.

**Table 3:** One example from the analytical process

Content	The post visually depicts one young man and two young women in a discussion. It also portrays a gender-inclusive workplace in the certified coaching activity. Age difference is not obvious
Production	The post links to the corporate policy stating the company’s commitment to diversity and expertise with individually customized coaching (as part of diversity discourse)
Use	A photograph displaying a young man at the center was chosen to post
Audiencing	One user commented on the photo, asking the agency to further promote diversity

## Results

Our study identified three ways that age and gender played out on the LinkedIn pages of staffing agencies, namely visualizing workplace diversity, (re-)producing divisions and specializations of labor, and creating employee ideals in a disembodied manner.

### Visualizing workplace diversity

The staffing agency diversity discourse identified in this study consisted of policies, programs, content posting practices, and lexical items in language. As indicated in the LinkedIn posts, all sampled staffing agencies had policies aimed at promoting a diverse and inclusive workplace culture, with these policies visually depicted. Additionally, these agencies provided consultation services to client companies regarding workforce diversity, while also depicting increasing workforce diversity as one of the key contributions they have to offer for client companies and the labor market. For example, R Agency partially ascribed higher profitability and productivity to an increased ability to attract and retain workforce diversity, making use of evidence of the positive impacts of diversity. This practice suggests an economic logic of managing diversity and a strong branding proposition for the business success of agencies and their clients. Both R Agency and M Agency shed light on the recruitment process, an area where they can advance diversity in the workplace and generate value for their services. In the data corpus, productivity growth is often emphasized as a valuable aspect of diversity discourse from a corporate perspective. This suggests that staffing agencies of inquiry scarcely noted the potential impacts that diversity discourse may have on personal and professional growth, such as enabling employees to develop capabilities to work with others and helping to ensure a qualified workforce.

As suggested by comments from various users sampled in this study, there is a divergence between diversity discourse and the ways that staffing agencies practice this discourse. Example 1 shows corporate diversity discourse discussed and challenged by a LinkedIn user who expressed the sentiment that staffing agencies insufficiently practice diversity. In such cases, staffing agencies may either reclaim their vision of cultivating a diverse workplace culture without commenting on a lack of diversity practices, or not respond to harsh critiques. Given that few LinkedIn posts by staffing agencies mention the ways in which they promote diversity or support disadvantaged groups, these posts seem decorative and not offering useful suggestions of fostering diversity in the workplace. In this sense, diversity loses its performativity as a concept and becomes a fashionable and marketable label.

#### Example 1

*Headline: 4 reasons why diversity works.*

*Visual image* *:* *One black woman looks up with a smiling face.*

*Caption: Diversity works. Not only because it’s the right thing to do, but because it’s good for business. Here’s why: [webpage link] #Diversity #Inclusion #Recruitment #DiversifyYourWorkforce #Diversity*

*Comments:*

– *It’s not working at all. (1 like, 2 replies)*
– *I’m sorry to hear you feel that way, but we believe a diverse and inclusive workplace is important and in everyone’s best interest. Hope you read the white paper. I’d love to hear your thoughts on it. (1 like, no reply)*
– *Clearly you were asked to post this… but this is not at all something [company’s name] supports. (2 likes, no reply).*

Diversity discourse shapes the ways that age and gender play out in visual media. Visual portrayals indicate multiple axes of social division covering age, gender and sexuality, race and ethnicity, and disabilities. Viewing gender as a social division, women are visually portrayed as coming from diverse backgrounds in terms of social categories like race, sexuality, and disabilities; men are visually represented with greater diversity in terms of age, race, and sexuality. Additionally, while the staffing agencies portrayed older male workers, they scarcely portrayed older women in their LinkedIn posts. Considering the media’s role in defining power relations, the invisibility of older women may serve to preserve the asymmetrical power structures disadvantaging them.

In their diversity discourse practice, staffing agencies portrayed a wide range of young women on particular occasions. For instance, on International Women’s Day, young women were mostly presented as having different work attitudes, values, aspirations, and beliefs (non-observable diversity), leading to the salience of the visual portrayals of women. As shown in Example 2, R Agency paid attention to women’s achievements and raised awareness of gender bias. As another example, young women had a visual prominence as the promoters/champions of diversity, equity, and inclusion (DEI) initiatives. For instance, they were portrayed as promoting a diverse workplace culture and challenging stereotypes of gender, sexuality, and disability. In this sense, young women are portrayed in more non-traditional roles and “decorative” images (c.f. Plakoyiannaki et al. 2008), which contests the hegemonic system of power relations based on traditional gender norms.

#### Example 2

*Headline: Happy International Women’s Day.*

*Visual image: A collage portrait of eight happy women.*

*Caption: Happy #InternationalWomensDay! Here’s to all the leaders, activists, innovators and change-makers working to build a more equal future #IWD2021. (102 likes, celebration, care. no comments)*

### (Re-)Producing divisions and specializations of labor

As indicated by the LinkedIn posts, staffing agencies have the ability to assign gendered occupation roles and create respective meanings. Specifically, the women in this study were mostly portrayed in female-gendered occupations (e.g., human resources, public relations, trainers). They were also depicted in activities of recruiting talents, sharing good practices for writing job descriptions, making the hiring process more transparent, re-skilling workers, adapting to digital transformation in the staffing sector, and leveraging online tools to improve staffing services. In particular, all the sample staffing agencies portrayed young women professionals as being experts in training staff on how to gain trust with employees, increase self-confidence, and achieve work-life balance. On the other hand, men were assigned to male-gendered occupations (e.g., engineers, construction workers) with power and knowledge handling management duties and undertaking physically demanding work. These portrayals of gender, as signifying practices, create gendered divisions and specializations of labor in the workplace. It should be noted that some photographs were stock images and were posted multiple times (see Example 3), reinforcing gendered occupations and specializations of labor.

#### Example 3

*Headline: Regulatory affairs trainee.*

*Visual image: A half-body shot showing a young woman smiling and sitting next to a table, and a textual description of “education in life sciences, attention to details & fast learner, fluent English, experience with SharePoint is an advantage”*

*Caption: Salesforce test #5. We are currently looking for a Regulatory Affairs Trainee. If you are interested in this role do not hesitate to contact our colleague XX. For more info: (link to website). (28 likes, 3 comments)*

In LinkedIn posts showing young women, they were portrayed as being the next generation of talents with relevant skills for the future, including critical thinking, emotional intelligence, and skills related to innovation and creativity, problem-solving, and collaboration. As posted by M Agency, early adulthood is a life phase where people “are ready to work” and have “a successful future.” Yet, such portrayals of women echo a gendered ideology of “youthfulness,” which is a dimension of gendered ageism (c.f. Spedale et al. 2014). As agencies overlook the opportunities and abilities that come from age, they may risk merely serving to benefit a small group that is already further ahead, while disregarding an aging workforce that is more likely to experience social inequality related to ageism. In this regard, young women are advantaged in terms of the status characteristic of age.

Within male-gendered portrayals, older men (mostly the young-old) were dominantly portrayed as chief executive officers or other senior professionals, positions which may be disproportionately held by men. Specifically, the R, M and K Agencies portrayed men as senior managers reaffirming a corporate commitment to advancing diversity, sharing insights about new trends in the global workforce (e.g., digitalization, the gig economy), and supporting clients to effectively deploy resources to the workforce. Such portrayals are associated with gender norms, such as more high-level responsibilities, challenging roles, and key leadership assignments, meaning that these men are advantaged in terms of the status characteristic of gender.

### Creating employee ideals in a disembodied manner

As shown in the LinkedIn posts, staffing agencies may disembody some workers of gender and age attributes in the workplace, primarily using two practical techniques. The first technique is de-contextualizing characters of different genders and ages in posts. This refers to the practice in which staffing agencies pair visuals with misleading LinkedIn captions. For instance, staffing agencies may use smiling portraits when promoting various purposes or agendas (e.g., showcasing work achievements, cultivating a positive corporate image, and creating a professional network). The second technique is to lose touch with the physicality of life. One type of LinkedIn photograph features partial- or half-body stock shots, representing people as being less socially present. These practices may lead to difficulties in distinguishing people’s gender and age, as they deprive viewers of the lived body of experience.

The practice of disembodying suggests an organizational logic that constructs a disembodied worker existing only for the work they are being associated with. Example 4 shows that the main female character is presented as a disembodied cluster of attributes (i.e., adaptive and effective leadership), which are available for inspection and possibly “taken on” by others. The visual design (a disembodied worker surrounded by a group of people) also conveys a sense of appraisal and creates employee ideals. One female LinkedIn user in the sample commented on how real-life experience can make it difficult to achieve the disembodied worker norm, which implies that the disembodied worker status may be more difficult for women than men to achieve.

#### Example 4

*Visual image: A young woman standing in front of a group of men that sit next to a table. The woman has her back turned to the readers.*

*Caption: According to experts, the most effective leaders adhere to situational leadership – a theory that states no single form of leadership is best, instead, the situations and tasks at hand define the required leadership style. Learn more – read “What is situational leadership? [webpage link] (82 likes, 10 comments)*

*Comments:*

– *Woman-User1: Great article. Unfortunately, many companies have no working knowledge or follow a situational leadership style. I think we can all relate at one time or another in our lives of having experience. (no like, 1 reply)*
– *K Agency’s reply: It’s always a work in progress. Some companies are further ahead than others for sure. (no like, no reply)*

## Discussion

This study explores the ways in which age and gender relations played out on the LinkedIn pages of global staffing agencies using an intersectionality lens. The results of this study indicate that corporate diversity discourse shapes the ways that different social groups are portrayed in the media, including by showing diverse groups and individual differences in several dimensions. Such portrayals may allow people to increasingly think about society and the workforce from the perspective of diversity. However, as questioned by the audience, the LinkedIn posts, especially visual images, may not actually promote practices that create and manage diversity and support disadvantaged groups to gain better employment opportunities. This echoes the macro-shift occurring within diversity discourse in the corporate sector from preventing discrimination to celebrating diversity (Vacchelli and Mesarič 2021). The practices of staffing agencies suggest that diversity risks being emptied from the initial call of fostering equal opportunities for those historically marginalized social categories, especially when diversity serves to promote the brand name of corporate entities. Additionally, celebrating diversity rhetorically may ease the tension embedded in a diverse workforce, allowing employers to overlook the power struggles that diversity entails.

On the question of how age and gender are portrayed (RQ1), this study found that young women were represented as being a new generation of leaders with certain individual differences. Older men (mostly the young-old) were portrayed in line with gender stereotypes (e.g., by showing male-gendered occupations and stereotypes), while older women were invisible.

Another important finding of this study is the complex way in which age and gender systems contributed to social inequality (RQ2). This may add to the existing literature on gendered ageism in the workplace by showing compound and intersectional forms of disadvantage/advantage. In this study, staffing agencies were found to harness the power of young women (age and gender), indicating a powerful age system and a diminishing gender system. Additionally, older men (mostly the young-old) were advantaged mostly in terms of the status characteristic of gender, suggesting a dominant gender system in the symbolic process. Moreover, this study found older male workers and young females to be portrayed as prestigious and influential, meaning that the invisibility of older women may be an outcome that gender and age systems jointly lead to. These findings broadly support the work of other studies in this area showing the dynamic relations between gender and age systems (e.g., Spedale et al. 2014; Wilińska 2010). In addition to the surfacing of age systems and/or gender systems, the results of this study indicate a special setting where age and gender relations are dissolved, i.e., disembodying workers.

**Fig. 2:**
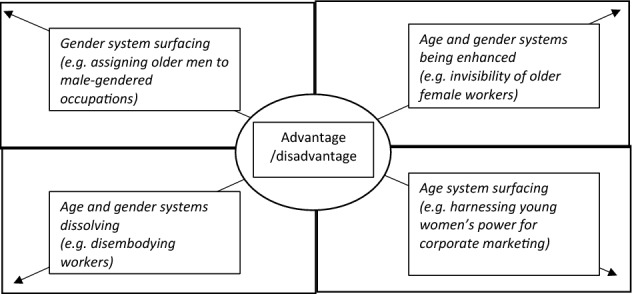
Compound and intersectional forms of disadvantage/advantage

Based on the findings, Fig. 2 displays the compound and intersectional forms of disadvantage/advantage in the study context, which may further the understanding of the social and symbolic process of age and gender systems.

One of the issues that emerges from the findings of this study concerns the implications of age and gender discourse in the media for individuals in real-life contexts. The naturalization of social inequalities is embedded in social institutions, which includes the media. The ways in which age and gender play out in the media may cause inequality in the workplace and throughout the course of people’s lives, given that age and gender relations interact to shape life situations and render disadvantages mostly for older women (e.g., a manifestation of gendered ageism). As argued by Cleveland et al. (2017), age and gender relations in the workplace can shape identities, which can in turn affect work perceptions and outcomes. For instance, a study focusing on a bank in Central America showed that gendered expectations inscribed in occupational roles can result in a variety of gender-unequal outcomes for workers and organizations (Doering and Thébaud 2017). Additionally, a Danish study analyzing discourses of sex/gender, age, power, and disciplinary position in academic contexts found that complex networks of discursive practices can transform pathways laid out for individuals in their lives (Søndergaard 2005). Furthermore, Beaton (2018) argued that social inequalities (e.g., experiences of being unfairly forced out and facing unjustified obstacles re-entering the workforce) can negatively affect older women’s economic stability, self-esteem, social connectedness, and emotional well-being. These findings provide support for the hypothesis that inequality at the intersection of age and gender may be created and reproduced by the media, thereby disregarding the powerless and serving the interests of the powerful.

The findings of this study have practical implications for staffing agencies in terms of developing diversity and inclusion (D&I) policies, as well as creating visual images portraying age and gender. To promote equal opportunities and fight discrimination, Trappel (2019) argued that the media should promote more positive and heterogenous portrayals of marginalized groups (e.g., women, older people, ethnic minorities). This study proposes that staffing agencies not only move beyond recognizing and representing more identity-based groups and individual differences in multiple ways, but also be more gender- and age-responsive. Specifically, staffing agencies should be aware of their impacts and modify their practices to actively foster a transformative change that prevents intersectional forms of discrimination and supports the professional growth of disadvantaged groups.

## Conclusions, limitations and implications for future research

This study investigated the ways that age and gender were represented on the LinkedIn pages of global staffing agencies through an intersectionality lens. It was found that diversity discourse shaped the ways that age and gender were represented, including by recognizing a greater number of identity groups and individual differences in various dimensions. The portrayals of age and gender were found to create gendered specializations of labor and reproduce gender stereotypes; additionally, some workers were represented as disembodied clusters of attributes. The results of this study illustrate the complex ways in which age and gender systems unfold, including mutually reinforcing, gender-/age-surfacing, and dissolving. The findings suggest that the portrayals may not support disadvantaged groups in terms of gaining access to better employment opportunities. This study proposes that staffing agencies actively address intersectional disadvantages and foster a gender- and age-transformative change.

The results of this analysis should be considered in light of certain limitations. Firstly, the sample is limited to five influential global staffing agencies, which is due to the study authors’ limited capability to manually retrieve data from LinkedIn. Despite this limited sample size, the study offers insights into gendered ageism and intersecting inequality that people may experience in the workplace, supported by qualitative data from the five largest global staffing agencies. There is a need for future research using a wider data corpus, including AI-retrieved big data which may highlight similar or additional power dynamics. Secondly, results are based on the authors’ own critical discourse analysis, which may be liable to issues of subjectivity. This was addressed by adequately discussing and collaboratively conducting the analysis. Given the positionality and analytical lens stated previously, this study focused on generating more nuanced views on the ways that age and gender systems play out on LinkedIn. Making use of this intersectionality lens, future qualitative and quantitative studies can explore intersecting inequality that people may experience in regard to age, gender, and other social divisions.

## Notes

All examples were transcribed into readable written text by the authors. Visual examples (the original format) are listed in the Appendix.

## Appendix

Example 1

Example 2

Example 3

Example 4
